# Enterococci in river Ganga surface waters: Propensity of species distribution, dissemination of antimicrobial-resistance and virulence-markers among species along landscape

**DOI:** 10.1186/1471-2180-9-140

**Published:** 2009-07-18

**Authors:** Pushpa Lata, Siya Ram, Madhoolika Agrawal, Rishi Shanker

**Affiliations:** 1 Environmental Microbiology Division, Indian Institute Toxicology Research (C.S. I.R.), Post Box 80, Mahatma Gandhi Marg, Lucknow-226001, U.P, India; 2 Department of Botany, Banaras Hindu University, Varanasi-221005, U.P, India

## Abstract

**Background:**

Surface waters quality has declined in developing countries due to rapid industrialization and population growth. The microbiological quality of river Ganga, a life-sustaining surface water resource for large population of northern India, is adversely affected by several point and non-point sources of pollution. Further, untreated surface waters are consumed for drinking and various household tasks in India making the public vulnerable to water-borne diseases and outbreaks. Enterococci, the 'indicator' of water quality, correlates best with the incidence of gastrointestinal diseases as well as prevalence of other pathogenic microorganisms. Therefore, this study aims to determine the distribution of species diversity, dissemination of antimicrobial-resistance and virulence-markers in enterococci with respect to rural-urban landscape along river Ganga in northern India.

**Results:**

Enterococci density (χ^2^: 1900, *df*: 1; *p *< 0.0001) increased from up-to-down gradient sites in the landscape. Species diversity exhibit significant (χ^2^: 100.4, *df*: 20; *p *< 0.0001) and progressive distribution of *E. faecalis*, *E. faecium*, *E. durans *and *E. hirae *down the gradient. Statistically discernible (*p*: 0.0156 – < 0.0001) background pool of resistance and virulence was observed among different *Enterococcus *spp. recovered from five sites in the up-to-down gradient landscape. A significant correlation was observed in the distribution of multiple-antimicrobial-resistance (viz., erythromycin-rifampicin-gentamicin-methicillin and vancomycin-gentamicin-streptomycin; *r*_*s*_: 0.9747; *p*: 0.0083) and multiple-virulence-markers (viz., *gelE*^+^*esp*^+^; *r*_*s*_: 0.9747; *p*: 0.0083; *gelE*^+^*efaA*^+^; *r*_*s*_: 0.8944; *p*: 0.0417) among different *Enterococcus *spp.

**Conclusion:**

Our observations show prevalence of multiple-antimicrobial-resistance as well as multiple-virulence traits among different *Enterococcus *spp. The observed high background pool of resistance and virulence in enterococci in river waters of populous countries has the potential to disseminate more alarming antimicrobial-resistant pathogenic bacteria of same or other lineage in the environment. Therefore, the presence of elevated levels of virulent enterococci with emerging vancomycin resistance in surface waters poses serious health risk in developing countries like India.

## Background

Enterococci, commensal organisms in gastrointestinal tract of human and animals have emerged as a leading cause of nosocomial infections [1]. *Enterococcus faecalis *(*E. faecalis*) and *E. faecium *are the two major pathogenic species in human, with sporadic infections caused by *E. durans*, *E. hirae *and other enterococci [2]. The presence of enterococci as an indicator of fecal contamination has been used in management of recreational water quality standards as it correlates best with the incidence of swimming-related illnesses [3,4]. Various virulence traits such as gelatinase (*gelE*), enterococcal surface protein (*esp*), collagen binding protein (*ace*) and endocarditis-associated antigen (*efaA*) have been considered as possible factors to play an important role in making enterococci a potential pathogen [5-7]. The enterococcal infections caused due to the potential virulence factors are difficult to treat because of the high level of intrinsic antimicrobial-resistance [8]. Several independent studies have reported the spread of antimicrobial-resistance and virulence-markers in clinical settings [2,9-13]. However, very little is known about the distribution of antimicrobial-resistance and virulence-markers among different species of enterococci from surface waters [14,15].

The surface waters in populous countries have become reservoirs of antimicrobial-resistant pathogenic microbes due to indiscriminate use of antimicrobials in human and veterinary medicine and addition of fecal contamination through point as well as non-point sources, storm drain infrastructure and malfunctioning septic trenches [16]. The propensity of species dissemination and prevalence of background level of antimicrobial-resistance is influenced by a variety of biotic and abiotic factors including geographical area and demography [17]. Recently, the presence of STEC (Shiga toxin producing *E. coli*) and ETEC (Enterotoxigenic *E. coli*) specific virulence genes and *E. coli *resistant to multiple antimicrobials has been reported from selected locations of river Ganga [18]. However, there is paucity of information on the concentration of enterococci and distribution of associated antimicrobial-resistance and virulence-markers in river Ganga. The river Ganga is a major river of Indian subcontinent traversing 2510 km across the country. The river and its tributaries provide 40% of water requirement of the country for various purposes including irrigation, daily use and drinking [19]. About 2460 million liters per day (mLd) of domestic sewage waste and 4570 mLd of raw sewage (from 223 cities and towns) directly finds its way into the river through its tributaries [20]. Further, other non-point sources include wastes from agriculture, health sector, practices of holy-dip and crematory processes along the banks.

The goal of current study was to contextualize the dissemination of species diversity, antimicrobial-resistance and virulence-markers in enterococci with respect to rural-urban landscape along river Ganga in northern India.

## Results and discussion

### Concentration of enterococci

**Figure 1:**
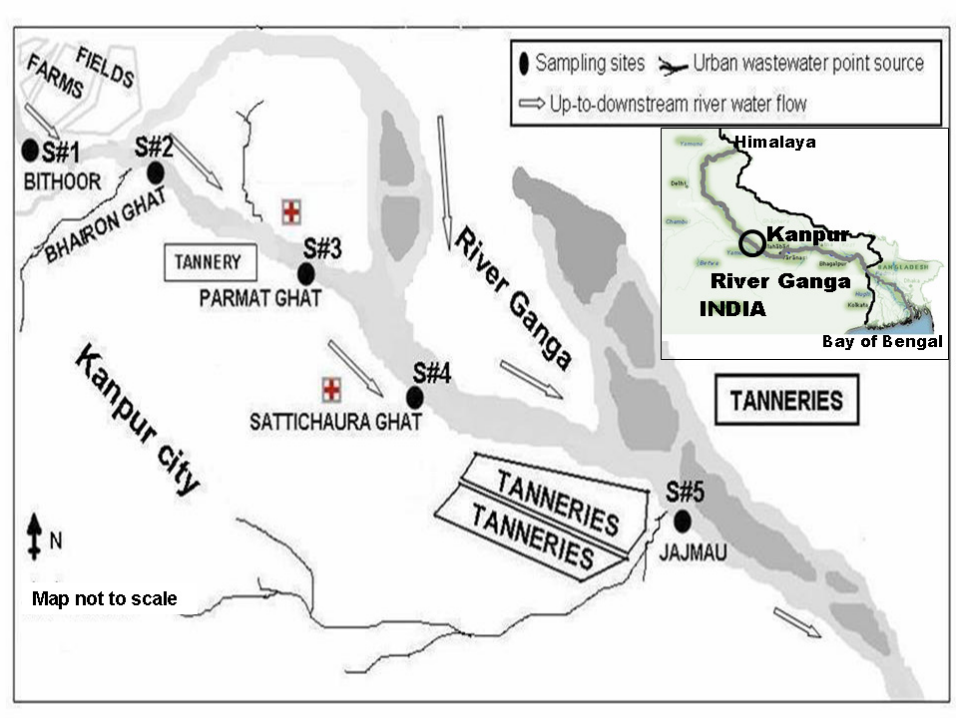
**Map of study area/sampling sites in the landscape**. Inset view simulates the complete 2510 km stretch of river Ganga from Himalaya to Bay of Bengal. Abbreviations: S#1, site 1: Bithoor (most upstream site); S#2, site 2: Bhairon ghat; S#3, site 3: Parmat ghat; S#4, site 4: sattichaura ghat or nana-rao ghat; S#5, site 5: jajmau (most downstream site). Arrows indicate the direction of surface water flow in the up-to-down-gradient fashion in the landscape. Topographic data based upon Survey of India map (adopted from http://www.ttkmaps.com).

Median concentrations of fecal streptococci or enterococci increased gradually and significantly (χ^2^: 1900, df: 1; *p *< 0.0001) in the river Ganga surface waters from up-to-down-gradient sites in the landscape (Table 1). The most downstream site was 53 and > 25000-fold more polluted than the most upstream site, using MPN test and membrane filtration method, respectively (Table 1). These observations concur with recent reports that determined the presence of fecal indicators in surface water gradients [18,21,22]. In the present study, we observed an increasing trend of enterococci concentration in the range of 2.3–4.4 × 10^1^CFU/100 mL, 1.0–1.2 × 10^3^CFU/100 mL, 6.7–7.7 × 10^4^CFU/100 mL, 4.4–5.1 × 10^4^CFU/100 mL and 7.8–8.7 × 10^5^CFU/100 mL at sites 1, 2, 3, 4 and 5 respectively (Figure 1). Internationally, the single-sample advisory limit of enterococci for fresh water is 61 CFU/100 mL and the 5-day geometric mean should not exceed 33 CFU/100 mL; while Indian standards do not delineate the limit for enterococci in terms of CFU/100 mL [3,19].

**Table 1:** Quantitative enumeration and density estimation of enterococci in surface water samples (*n *= 15) collected from sites (*n *= 5) located on river Ganga (Kanpur city) in up-to-down-gradient fashion

Sampling Site	CFU/100 ml water [Median (Range)]	MPN index/100 ml water (Lower 95% CI – Upper 95% CI)^a^	*p*-Value^b^
Site 1	32 (23 – 44)	30 (10 – 110)	
Site 2	1130 (1034 – 1211)	220 (100 – 580)	
Site 3	73000 (67532 – 76848)	350 (160 – 820)	< 0.0001***
Site 4	48000 (43978 – 51078)	300 (100 – 1300)	
Site 5	820000 (782841 – 871978)	1600 (600 – 5300)	
Control^c^	ND	ND	

^a^Lower 95% CI – Upper 95% CI are adopted from Table 9221.IV, Section 9221C. Estimation of Bacterial density, APHA (1998). ^b^*p*-Value was calculated using chi square test for trend, χ^2 ^= 1900; df = 1. ^c^Sterile Milli Q water used as control. ***Statistically significant at alpha < 0.05.

Abbreviations: ND, Not Detected.

### *Enterococcus* spp. isolated from river Ganga waters

A significant (χ^2^: 100.4, df: 20; *p *< 0.0001) heterogeneity and diversity was observed in *Enterococcus *spp. recovered from river Ganga surface water samples collected from five different sites (Table 2). The spatial heterogeneity of *Enterococcus *spp. varied widely along the landscape, depending upon exposure to various environmental and anthropogenic factors. In general, the enterococcal spatial heterogeneity seems to be introduced either via point sources (urban sewage, clinical and industrial discharge) or nonpoint sources (agricultural runoff and storm-water route).*E. faecalis *(64%) was found to be the most prevalent species followed by *E. faecium *(24%) throughout the landscape. A gamut of factors appears to complement the increase of *E. faecalis *and *E. faecium *coexistence towards the down-gradient sites in the similar environmental niche. The coexistence of these two genotypes in one niche may be due to their differential affinity and efficiency of resource utilization complementing similar phenomenon reported elsewhere for *Vibrio cholerae *serogroups; O139 Bengal and O1 E1 Tor [23]. In the same study, the enhanced affinity of *V. cholerae *O1 E1 Tor to colonize copepods was observed to be a contributory factor for its dominance in cholera epidemic. Likewise *E. faecalis*, the most prevalent species observed in this study has been implicated in *ca*. 67% and 90% of enterococcal infection cases associated with multiple-antimicrobial-resistance in different clinical studies conducted in India and USA respectively [12,24]. *E. durans *and *E. hirae *were not evenly distributed throughout the landscape. The presence of *E. hirae *(2%) was observed only at the locations which receive tannery effluents contaminated with heavy metals. The prevalence of *E. durans *(8%) appears to be affected by urban wastewater point-source contamination. The "other *Enterococcus *spp." was present at site 5 only. Moreover, it appears that the environmental factors account for the spatial variation of *Enterococcus *spp. in the landscape.

**Table 2:** Frequency of distribution of *Enterococcus *spp. diversity among sites (*n *= 5)

Sampling Site	No. of isolates (%)						*p*-Value
		
	*E. faecalis*	*E. faecium*	*E. durans*	*E. hirae*	other *Enterococcus *spp.	Total enterococci per site	
site 1	5 (5.95)	1 (1.19)	0	0	0	6 (7.14)	
site 2	9 (10.71)	4 (4.76)	4 (4.76)	0	0	17 (20.24)	
site 3	12 (14.29)	6 (7.14)	0	1 (1.19)	0	19 (22.62)	
site 4	12 (14.29)	3 (3.57)	3 (3.57)	0	0	18 (21.43)	<0.0001***
site 5	16 (19.05)	6 (7.14)	0	1 (1.19)	1 (1.19)	24 (28.57)	
*Enterococcus *spp. distribution	54 (64.29)	20 (23.81)	7 (8.33)	2 (2.38)	1 (1.19)	84 (100)	

^a^*p*-Value was calculated using chi square test, χ^2 ^= 100.4; df = 20. ***Statistically significant at alpha < 0.05.

### Antimicrobial-resistance

This study investigated the background pool of antimicrobial-resistance (BPAR) in the landscape. High frequency of multiple-antimicrobial-resistance (MAR) was recorded among enterococci tested. The number (median) of antimicrobials against which resistance was observed in each *Enterococcus* isolate increased significantly (*p *0.0156, 0.0001, < 0.0001, 0.0001, < 0.0001) towards downstream in the landscape (Table 3). The prevalence of resistance to a minimum of five antimicrobials per isolate reflects high BPAR in the up-to-down gradient landscape. This high value of BPAR at most upstream site could be attributed to the agriculture farms, intensive livestock and swine farming in the locality. Although there is no data available from India, the prevalence of VRE on site 1 may be due to the use of antimicrobials in the animal feed and cattle or swine manure application in the fields, reported to be important contributing factors elsewhere [25,26]. The urban sewage waste contributed to the maintenance of resistance pool at site 2. The elevated level of resistance at site 3 was a likely contribution from hospital, tannery, and sewage discharging point sources leading to microbial, chemical as well as antimicrobial contamination. The lower concentration of enterococci and reduced resistance pool at site 4, as compared to site 3, is possibly due to confluence of two watersheds just upstream of site 4 resulting in dilution of the pre-existing microbial biogeography and associated traits. Site 5, the most downstream sampling station in the landscape presents the worst scenario of microbial contamination and reflects the best spatial correlation among enterococci concentration, species diversity, antimicrobial-resistance and virulence-markers' dissemination.

**Table 3:** Antimicrobial-resistance and virulence-markers investigated in each *Enterococcus* isolate on sites located in the up-to-down-gradient landscape

Sampling site	No. of samples analyzed for antimicrobial susceptibility or virulence-marker/s (%)	Antimicrobial-resistance (AR) and Virulence-markers (VM) characterized per isolate [Median (Range)]^a^	*p*-Value^b^
site 1	6 (7.14)	AR: 5 (3 – 6)	0.0156*
		VM: 2 (1 – 3)	0.0156*
site 2	17 (20.24)	AR: 5 (4 – 5)	0.0001*
		VM: 2 (1 – 2)	0.0002*
site 3	19 (22.62)	AR: 7 (5 – 7)	< 0.0001*
		VM: 1 (1 – 4)	< 0.0001*
site 4	18 (21.43)	AR: 5 (5 – 6)	0.0001*
		VM: 2 (1 – 2)	< 0.0001*
site 5	24 (28.57)	AR: 5 (5 – 6)	< 0.0001*
		VM: 2 (2 – 3)	< 0.0001*

^a^Median and range are reported to match more consistently with the nonparametric statistical tests performed.

^a^*p*-Value was calculated using Wilcoxon rank sum test. *Statistically significant at alpha = 0.05.

**Figure 2:**
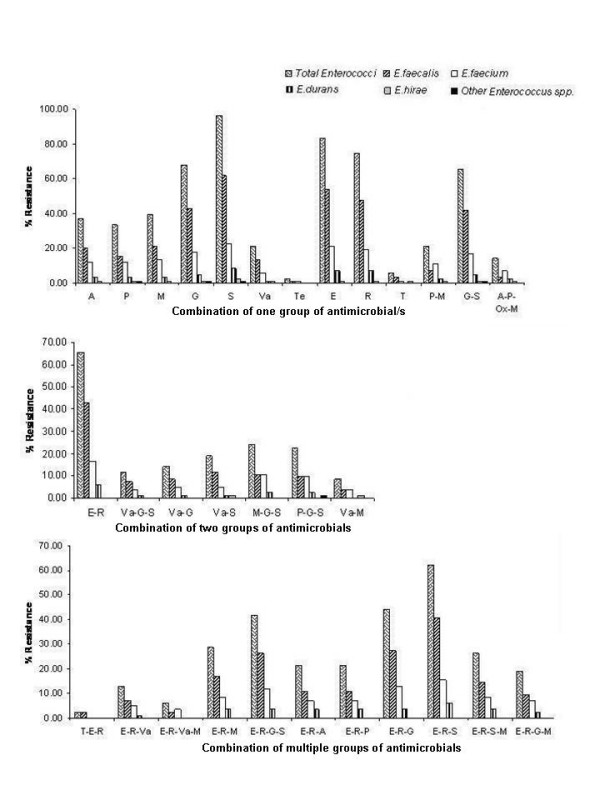
**Distribution of single/multiple-antimicrobial-resistance in different *Enterococcus *spp**. Abbreviations: A, ampicillin; P, penicillinG; M, methicillin; G, gentamicin; S, streptomycin (aminoglycoside); Va, vancomycin (glycopeptide); Te, teicoplanin; E, erythromycin; R, rifampicin; T, tetracycline; P-M, penicillinG-methicillin; A-P-Ox-M, ampicillin-penicillinG-oxacillin-methicillin (β-lactam); E-R, erythromycin-rifampicin (Macrolide-rifamycin); Va-G-S/Va-S/Va-G (glycopeptide-aminoglycoside); M-G-S/P-G-S (β-lactam-aminoglycoside); Va-M (glycopeptide-β-lactam); T-E-R (tetracycline-macrolide-rifamycin); E-R-Va (macrolide-rifamycin-glycopeptide); E-R-Va-M (macrolide-rifamycin-glycopeptide-β-lactam); E-R-M/E-R-A/E-R-P (macrolide-rifamycin-β-lactam); E-R-G/E-R-S (macrolide-rifamycin-aminoglycoside); E-R-S-M/E-R-G-M (macrolide-rifamycin-aminoglycoside-β-lactam). All antimicrobial combinations derived from aforementioned antimicrobial abbreviations.

Statistically detectable MAR was recorded among *Enterococcus *spp. isolates [Figure 2 and Additional file 1]. *E. faecium *resistant to β-lactam class of antimicrobials including methicillin was recorded to be higher in this landscape. A large scale dissemination of aminoglycoside resistance was observed along the landscape gradient; higher percentage of gentamicin resistant enterococci were prevalent at site 3 which reflects its frequent use in human medicine as this site receives wastes from hospital located just upstream. Our observations indicate that streptomycin and gentamicin resistance are distributed extensively in the environmental gene pool. The resistance to erythromycin, a macrolide and rifampicin in association with vancomycin, a glycopeptide was also distributed significantly. Hasman et al. [27], reported a relationship between copper, glycopeptide and macrolide resistance among *E. faecium *strains isolated from pigs in Denmark during 1997–2003, contemplating persistence of BPAR in that geographic region. A number of studies have reported the phenomenon of sustained BPAR in poultry and local population [28,29].

Though the frequency of VRE is only 21% in the landscape, its association with other widely disseminated antimicrobials and virulence determinants may lead to evolution of pathogenic VRE and thus reduce the chances for synergistic therapy in case of failure of single antimicrobial [30]. Recently, Lata et al. [31] reported the prevalence of *vanA *gene (for vancomycin resistance) in surface waters of river Ganga and its tributary and discussed the possible consequences of BPAR, its environmental carriage by plasmid maintenance systems or postsegregational killing (PSK) systems.

### Dissemination of virulence-markers

This study also explored the dynamic and complex facet of landscape and its effects on the dissemination of virulence-markers *gelE*, *ace*, *efaA *and *esp *among different *Enterococcus *spp. Statistically discernible distribution of virulence-markers along the up-to-down-gradient landscape was observed (Table 3). In addition, the active gelatinase phenotype was observed in 19.05% *E. faecalis *isolates [see Additional file 2]. The background level of virulence-markers in the up-to-down gradient landscape exist at least for two virulence-markers predominantly *gelE*^+^*esp*^+ ^(26.19%) followed by *gelE*^+^*efaA*^+ ^(7.14%). The only exception was site 3 with median value of one which otherwise exhibited the range of one to four virulence-markers *gelE*^+^*efaA*^+^, *gelE*^+^*efaA*^+^*esp*^+^,*gelE*^+^*ace*^+^*efaA*^+ ^and *gelE*^+^*ace*^+^*efaA*^+^*esp*^+^. The impact of landscape and associated environmental factors seem to affect the dissemination of all four virulence-markers at site 3 which receives contamination from hospital wastes, municipal sewage and tannery effluents. Enterococci isolates from the most polluted downstream site exhibited a range of two to three virulence-markers per isolate; *gelE*^+^*esp*^+ ^and *gelE*^+^*efaA*^+^*esp*^+ ^combinations were the most prevalent multiple-virulence-traits.

Significantly, the correlation of four virulence-markers was identified either singly or in combination with *Enterococcus *spp. diversity from river Ganga surface waters (Table 4). Earlier reports on dissemination of virulence-markers in different enterococci suggest virulence-markers are common trait in the genus *Enterococcus*[7,32-34]. A recent study has reported the prevalence of gelatinase phenotype of enterococci in agricultural environment and suggested it as reservoir of clinically relevant strains [35]. The pervasiveness of virulence-markers investigated in the current study may be due to the evolution of pathogenic enterococci by natural conjugation in environmental waters that receive potential pathogenic enterococci from various point and non-point sources including urban land use, agriculture, intensive livestock operations, hospital and industrial wastes. The natural processes are too complex to comprehend although the transconjugation experiments conducted elsewhere demonstrated *in vitro *transfer of additional virulence determinants from clinical strains to starter strains [7]. In the present study, the phenotypic assay for gelatinase activity revealed that certain *E. faecalis *and different *Enterococcus *spp. isolates contained apparently silent *gelE *determinant. This observation is supported by an earlier report on presence of silent *gelE *gene possibly due to inactive gene product or down regulation of gene expression influenced by various environmental factors resulting in lack of phenotypic activity [7]. Further, the activation of silent genes by temporal factors existing in our body, the response of other commensal microbes in the gastrointestinal tract and the persistent presence of large numbers of preexisting commensal enterococci cannot be ignored. Our observation on the loss of gelatinase activity by sub-culturing is also supported by other studies reporting similar phenomena [7,35].

The coselection of resistance to vancomycin, methicillin, gentamicin, streptomycin and ciprofloxacin with *gelE *virulence-marker was observed in the landscape [see Additional file 2]. An *E. faecium *isolate was observed with resistance to gentamicin and MAR to vancomycin, erythromycin and rifampicin along with *gelE*^+^*efaA*^+^*esp*^+ ^virulence-determinants. The notoriety of *E. faecium *strains with multiple-antimicrobial-resistance especially VRE in debilitating the disease conditions is well established [10]. The combination of virulence-traits cytolysin-aggregation substance has been demonstrated to be highly coevolved and is efficiently transferred to the sensitive recipients by conjugation [36]. On the other hand a clinical strain of *E. faecium *having a conjugative plasmid, highly related to pCF10 of *E. faecalis*, has been shown to confer transferable high-level vancomycin resistance via conjugation [37]. These evidences indicate the possible transfer of linked virulence-traits and antimicrobial-resistance viz., vancomycin resistance in the landscape. Further the persistence of VRE in the environment even in the absence of antimicrobial selection pressure has been attributed to multiple types of PSK systems or Toxin-Antitoxin (TA) systems [28,38,39]. Though till date no role has been assigned to TA systems with respect to linked traits like multiple-antimicrobial-resistance and multiple-virulence-markers in VRE; it is possible that such systems might be playing pivotal role in persistence and dissemination of perilous antimicrobial-resistant pathogenic enterococci.

**Table 4:** Correlation observed for the prevalence of single/multiple-virulence-markers along with *Enterococcus *spp. diversity in the landscape.

		No. of isolates (%)							
				
S. No	Combination of virulence-marker/s	Total enterococci	*E. faecalis*	*E. faecium*	*E. durans*	*E. hirae*	Other *Enterococcus *spp.	Spearman correlation (*r*_*s*_)	*p*-Value^a^
1	*gelE*^+^	30(35.71)	17(20.24)	8(9.52)	3(3.57)	1(1.19)	1(1.19)	1	0.0083^**^
2	*esp*^+^	4(4.76)	0	2(2.38)	1(1.19)	1(1.19)	0	1	0.0083^**^
3	*efaA*^+^	4(4.76)	1(1.19)	2(2.38)	0	1(1.19)	0	0.8208	0.0667
4	*ace*^+^	2(2.38)	1(1.19)	0	0	1(1.19)	0	0.4472	0.225
5	*gelE*^+^*esp*^+^	22(26.19)	17(20.24)	2(2.38)	3(3.57)	0	0	0.9747	0.0083^**^
6	*gelE*^+^*efaA*^+^	6(7.14)	4(4.76)	2(2.38)	0	0	0	0.8944	0.0417^*^
7	*gelE*^+^*ace*^+^*efaA*^+^	2(2.38)	2(2.38)	0	0	0	0	0.7071	0.1167
8	*gelE*^+^*efaA*^+^*esp*^+^	15(17.86)	10(11.9)	4(4.76)	0	1(1.19)	0	0.8208	0.0667

^a^*p*-Value was calculated using Wilcoxon matched pair test. **/* *p*-value summary for significantly effective pairing.

## Limitations

Pitfalls and associated plausible explanations of this study concern (i) conventional culture based methodology opted for isolation of environmental strains; influenced by the dominance of culturable bacteria and does not consider viable but non-culturable (VBNC) entities which carry potential pathogenic and MAR traits to impart serious infections. The molecular metagenome based approach has been taken into account for our ongoing studies to overcome the limitation. (ii) Limiting landscape to a small geographic region due to financial constrains; consequently the most upstream location in the landscape does not hold the merit of pristine location to be considered for absolute estimation of background level or pool of resistance or virulence-determinants, only relative estimation of background level of resistance is the feasible option. More collaboration between the national and international labs is needed for the purpose. (iii) Lack of exact data on usage pattern of antimicrobials in human and veterinary medicine which further limits the study as the quantitative nature of cause-effect relationship remains partially explored. Strict rule codes needed to be set and maintained by the regulatory agency for local counterparts to keep the track record of supply as well as nature and mode of consumption. However, the intricacies in retrieving specific antimicrobial usage data based on individual consumption continue to be a global challenge for environmental health researchers in the absence of national and or state regulations that require consumers to report their consumption to the local authority as earlier mentioned by Sapkota et al [22].

## Conclusion

In the present study, the spread of potential pathogenic enterococci appears to be the manifestation of complex network of ecological processes and associated factors in the landscape of river Ganga. Enterococci recognized as hardy and rogue microbe may cause very serious infections with limited options of treatment. Surface waters with emerging VRE and background pool of multiple-antimicrobial-resistant and multi-virulent enterococci can contribute to the dissemination of resistance and virulence-determinants in the diverse *Enterococcus *spp. and other bacteria. Therefore, the presence of antimicrobial-resistant pathogenic enterococci in surface waters of populous nations demand improved surveillance for risk assessment and pre-emptive strategies for protection of public health.

## Methods

### Study site

The study was performed along 30 km landscape in and around Kanpur city (geographic coordinates: 26.4670° North and 80.3500° east, area: 1600 km^2^, estimated population: 4,864,674) located on the banks of river Ganga in up-to-down-gradient fashion (Figure 1). The most upstream Site 1 is Bithoor, a rural area with agricultural farms located 20 km upstream of the city. Site 2 is Bhairon ghat, it receives municipal waste from the locality. Site 3 is Parmat ghat, receives contamination through urban sewage, hospital and one tannery located upstream to it. Site 4 is Sattichaura ghat and two watersheds of river Ganga confluence just upstream of this site. Site 5 is Jajmau, the most downstream site, hub for tanneries and receives municipal waste from whole city.

### Sample collection

A cross-sectional approach was used to collect surface water samples. Samples were collected in triplicate (n = 15) from five locations situated in up-to-down-gradient fashion (Figure 1). In brief, three transects were established randomly at each site and water samples (1 L) were collected 30 cm below water surface from left, mid and right bank of the river along each transect. Surface water samples were stored in sterile glass bottles, labeled and transported on ice to the laboratory for analysis. Sample processing and analysis was conducted within 6 hr after sample collection.

### Isolation and enumeration of Enterococci

Quantitative enumeration of enterococci from selected sites was performed as per APHA [40] using the Multiple Tube Fermentation Technique and reported as MPN index/100 ml surface water. Additionally, enterococci were enumerated from each sample using standard membrane filtration method and reported as CFU/100 ml surface water [41]. Presumptive enterococci recovered (n = 30) from each sample were identified by biochemical tests including catalase test and PYR test. The growth of isolates was determined in 6.5% NaCl, pH 9.6, and at 10 and 45°C, respectively. All confirmed enterococci isolates were archived in tryptic soy broth with 15% glycerol at -80°C for further analyses.

### Characterization of *Enterococcus* spp. using Polymerase Chain Reaction

All isolates confirmed by biochemical tests were subjected to genotypic characterization by Polymerase Chain Reaction (PCR) technique. The presence of *tuf *gene encoding the elongation factor EF-Tu in genus *Enterococcus* and the *sodA *variant for *E. faecalis*, *E. faecium*, *E. durans *and *E. hirae *species were investigated by PCR as reported earlier [42,43]. An isolate not belonging to the four species of enterococci genotypically characterized by PCR in this study was listed as "other *Enterococcus *spp."

### Antimicrobial susceptibility testing

A panel of thirteen antimicrobials (antimicrobial abbreviation:mcg/disc) impregnated on paper discs (Himedia Ltd., India) belonging to eight different group of antimicrobials as Fluoroquinolone: Norfloxacin (Nx:10 mcg), β-lactam: Ampicillin (A:10 mcg), Oxacillin (Ox:1 mcg), PenicillinG (P:10 units), Methicillin (M:5 mcg), Aminoglycoside: Gentamicin (G:10 mcg), Streptomycin (S:10 mcg), Tetracycline: Tetracycline (T:30 mcg), Phenicol: Chloramphenicol (C:30 mcg), Macrolide: Erythromycin (E:15 mcg), Rifamycin: Rifampicin (R:5 mcg), Glycopeptides: Vancomycin (Va:30 mcg), Teicoplanin (Te:30 mcg) were used for testing the sensitivity of isolated organisms by Kirby-Bauer disc diffusion test as described by CLSI [31,44]. The diameter of zones showing inhibition were measured to the nearest mm and recorded. A zone size interpretive chart was used to determine sensitivity/resistance of antimicrobials as described by CLSI [44].

### Determination of virulence-markers distribution in enterococci

Polymerase Chain Reaction technique was used to generate a profile for virulence-markers' distribution in enterococci. The presence of genes *gelE*, *ace*, *efaA *and *esp *encoding gelatinase, adhesion collagen factor, endocarditis factor antigen and enterococcal surface protein, respectively in different *Enterococcus *spp. was examined by PCR as reported earlier [7,45]. The amplicons were electrophoresed on 2% agarose gel in Tris-acetate-EDTA buffer supplemented with 0.5 μg/ml of ethidium bromide and calibrated using 50 bp and 100 bp DNA ladders (MBI Fermentas, USA). All enterococci isolates were subjected to phenotypic gelatinase assay as described by Gilmore et al. [2]. *E. faecalis *ATCC 51229, *E. faecium *ATCC 35667, 27270, *E. durans *ATCC 49470, *E. hirae *ATCC 9790 were used throughout the study as reference/standard strains.

### Statistical analyses

We compared concentrations of enterococci obtained using MPN analysis test and membrane filtration method from up-to-down-gradient surface water samples. Chi-square test for trend was applied for the purpose. The distribution of *Enterococcus *spp. and its association with the landscape was evaluated using Chi-square test. The prevalence and distribution of antimicrobial-resistance and virulence-markers among isolates from up-to-down-gradient landscape was assessed using Wilcoxon rank-sum tests. Wilcoxon matched pair test was conducted to investigate correlation between dissemination of antimicrobial-resistance and virulence-markers in different *Enterococcus *spp. All statistical analyses were performed using GraphPad Prism version 5.0 for Windows (GraphPad Software, San Diego, California, USA, http://www.graphpad.com).

## Authors' contributions

PL carried out the phenotypic antimicrobial susceptibility profiling, determination of gelatinase activity, PCR characterization for determination of enterococcal species diversity and virulence marker's distribution, analysed the data and wrote the manuscript. SR contributed to sample collection and microbiological analysis. MA provided direction on available means of data analyses. RS conceived the study, analysed the data and wrote the manuscript. All authors contributed to the general content and structure of the final manuscript.

## Supplementary Material

Additional file 1 **Table A1- Correlation observed between the prevalence of single/multiple-antimicrobial-resistance and *Enterococcus* species diversity in the landscape**. Presentation of correlation between the single or multiple-antimicrobial-resistance and different *Enterococcus *species recovered from the landscape. Click here for file

Additional file 2 **Table A2- Site wise elaborated profile of species diversity, antimicrobial-resistance and virulence-markers in enterococci isolates from river Ganga at Kanpur city**. Depiction of investigated enterococcal species diversity, antimicrobial-resistance and virulence-markers' profile of all isolates recovered from the landscape. Click here for file
