# Supplementary material for: Enterococci in river Ganga surface waters: Propensity of species distribution, dissemination of antimicrobial-resistance and virulence-markers among species along landscape
Source: BMC Microbiol. 2009 Jul 18;9:140. doi: 10.1186/1471-2180-9-140 (PMC2722665; doi:10.1186/1471-2180-9-140)
Supplement: Additional file 1 — Table A1- Correlation observed between the prevalence of single/multiple-antimicrobial-resistance and Enterococcus species diversity in the landscape. Presentation of correlation between the single or multiple-antimicrobial-resistance and different Enterococcus species recovered from the landscape. [file 1471-2180-9-140-S1.doc]

**Additional Files**

## Enterococci in river Ganga waters: Propensity of species distribution, dissemination of antimicrobial-resistance and virulence-markers among species along landscape

## *Pushpa Lata^1^, Siya Ram^1^, Madhoolika Agrawal^2^ and Rishi Shanker^1*^*

*^1^Environmental Microbiology Division, Industrial Toxicology Research Centre* (C.S.I.R.),

## *Post Box 80, Mahatma Gandhi Marg, Lucknow-226001, U.P., India; ^2^Department of Botany, Banaras Hindu University, Varanasi-221005, U.P., India.*

**Corresponding author:* Dr Rishi Shanker

Phone: 91+ 0522 –2613786/2614118/2627586 Extn.237,

Fax : 91+ 0522-2611547,

e-mail : [rishishanker@gmail.com](mailto:rishishanker@gmail.com)/rishi@iitr.res.in

.

**Table A1.** Correlation observed between the prevalence of single/multiple-antimicrobial-resistance and enterococcus species diversity in the landscape.

| S. No. | Combination of single/multiple antimicrobial-resistance (group or class) | No. of total enterococci (%) | Spearman correlation (*r_s_*) | *p-*Value^a^ |
| --- | --- | --- | --- | --- |
| 1 | A (β-lactam) | 31 (36.90) | 1 | 0.0083**** |
| 2 | P (β-lactam) | 27 (32.14) | 0.9747 | 0.0083**** |
| 3 | M (β-lactam) | 33 (39.29) | 1 | 0.0083**** |
| 4 | G (aminoglycoside) | 56 (66.67) | 0.9747 | 0.0083**** |
| 5 | S (aminoglycoside) | 80 (95.24) | 1 | 0.0083**** |
| 6 | Va (glycopeptide) | 18 (21.43) | 0.9747 | 0.0083**** |
| 7 | Te (glycopeptide) | 2 (2.38) | 0.866 | 0.0417*** |
| 8 | E (macrolide) | 70 (83.33) | 1 | 0.0083** |
| 9 | R (rifamycin) | 63 (75) | 1 | 0.0083**** |
| 10 | T (tetracycline) | 5 (5.95) | 0.7379 | 0.0667 |
| 11 | P-M (β-lactam) | 18 (21.43) | 0.9 | 0.0417*** |
| 12 | G-S (aminoglycoside) | 55 (65.48) | 0.9747 | 0.0083**** |
| 13 | A-P-Ox-M (β-lactam) | 12 (14.29) | 0.9 | 0.0417*** |
| 14 | E-R (macrolide-rifamycin) | 55 (65.48) | 0.9747 | 0.0083**** |
| 15 | Va-G-S (glycopeptide-aminoglycoside) | 10 (11.90) | 0.9747 | 0.0083**** |
| 16 | Va-G (glycopeptide-aminoglycoside) | 12 (14.29) | 0.9747 | 0.0083**** |
| 17 | Va-S (glycopeptide-aminoglycoside) | 16 (19.05) | 0.9747 | 0.0083**** |
| 18 | M-G-S (β-lactam-aminoglycoside) | 20 (23.81) | 0.9487 | 0.0083**** |
| 19 | P-G-S (β-lactam-aminoglycoside) | 18 (21.43) | 0.8721 | 0.0417*** |
| 20 | Va-M (glycopeptide- β-lactam) | 7 (8.33) | 0.7906 | 0.0667 |
| 21 | T-E-R (tetracycline-macrolide-rifamycin) | 2 (2.38) | 0.7071 | 0.1167 |
| 22 | E-R-Va (macrolide-rifamycin-glycopeptide) | 11 (13.10) | 0.9747 | 0.0083**** |
| 23 | E-R-Va-M (macrolide-rifamycin-glycopeptide-β-lactam) | 5 (5.95) | 0.7826 | 0.0667 |
| 24 | E-R-M (macrolide-rifamycin-β-lactam) | 24 (28.57) | 0.9747 | 0.0083**** |
| 25 | E-R-G-S (macrolide-rifamycin-aminoglycoside) | 35 (41.67) | 0.9747 | 0.0083**** |
| 26 | E-R-A (macrolide-rifamycin-β-lactam) | 18 (21.43) | 0.9747 | 0.0083**** |
| 27 | E-R-P (macrolide-rifamycin-β-lactam) | 18 (21.43) | 0.9747 | 0.0083**** |
| 28 | E-R-G (macrolide-rifamycin-aminoglycoside) | 37 (44.05) | 0.9747 | 0.0083**** |
| 29 | E-R-S (macrolide-rifamycin-aminoglycoside) | 52 (61.90) | 0.9747 | 0.0083**** |
| 30 | E-R-S-M (macrolide-rifamycin-aminoglycoside-β-lactam) | 22 (26.19) | 0.9747 | 0.0083**** |
| 31 | E-R-G-M (macrolide-rifamycin-aminoglycoside-β-lactam) | 16 (19.05) | 0.9747 | 0.0083**** |

^a^*p-*Value was calculated using Wilcoxon matched pair test. **/* *p*-value summary for significantly effective pairing.
