# Supplementary material for: Enterococci in river Ganga surface waters: Propensity of species distribution, dissemination of antimicrobial-resistance and virulence-markers among species along landscape
Source: BMC Microbiol. 2009 Jul 18;9:140. doi: 10.1186/1471-2180-9-140 (PMC2722665; doi:10.1186/1471-2180-9-140)
Supplement: Additional file 2 — Table A2- Site wise elaborated profile of species diversity, antimicrobial-resistance and virulence-markers in enterococci isolates from river Ganga at Kanpur city. Depiction of investigated enterococcal species diversity, antimicrobial-resistance and virulence-markers' profile of all isolates recovered from the landscape. [file 1471-2180-9-140-S2.doc]

**Additional Files**

## Enterococci in river Ganga waters: Propensity of species distribution, dissemination of antimicrobial-resistance and virulence-markers among species along landscape

## *Pushpa Lata^1^, Siya Ram^1^, Madhoolika Agrawal^2^ and Rishi Shanker^1*^*

*^1^Environmental Microbiology Division, Industrial Toxicology Research Centre* (C.S.I.R.),

## *Post Box 80, Mahatma Gandhi Marg, Lucknow-226001, U.P., India; ^2^Department of Botany, Banaras Hindu University, Varanasi-221005, U.P., India.*

**Corresponding author:* Dr Rishi Shanker

Phone: 91+ 0522 –2613786/2614118/2627586 Extn.237,

Fax : 91+ 0522-2611547,

e-mail : [rishishanker@gmail.com](mailto:rishishanker@gmail.com)/rishi@iitr.res.in

.

**Table A2.** Site wise elaborated profile of species diversity, antimicrobial-resistance and virulence-markers in enterococci isolates from river Ganga at Kanpur city

| Sites | Isolates | *Enterococcus species distribution* | | | | | *Distribution of Antimicrobial agent resistance* | | | | | | | | | | | | | Distribution of virulence markers | | | |
| --- | --- | --- | --- | --- | --- | --- | --- | --- | --- | --- | --- | --- | --- | --- | --- | --- | --- | --- | --- | --- | --- | --- | --- |
|  |  |  |  |  |  |  | *1* | *2* | *3* | *4* | *5* | *6* | | *7* | | | | *8* | |  |  |  |  |
|  |  | *fl* | *fm* | *du* | *hr* | *Other* | *Cf* | *T* | *C* | *E* | *R* | *S* | *G* | *P* | *A* | *M* | *Ox* | *Va* | *Te* | *gelE* | *ace* | *efaA* | *esp* |
| site 1 | 2R1C | + |  |  |  |  |  |  |  | + |  | + | + |  | + | + | + |  |  | + |  | + | + |
|  | 2R1B | + |  |  |  |  |  |  |  | + |  | + |  |  |  |  | + |  |  |  |  | + |  |
|  | 2M1E | + |  |  |  |  |  |  |  | + | + | + |  |  |  |  | + |  |  | + |  |  | + |
|  | 2M1D | + |  |  |  |  |  |  |  | + |  | + | + |  |  |  |  | + | + | + |  |  | + |
|  | 2M1C | + |  |  |  |  |  |  |  | + | + | + | + | + | + |  |  | + |  | + |  |  | + |
|  | 2M1B |  | + |  |  |  |  |  |  | + | + |  | + |  |  |  |  | + |  | + |  | + | + |
| site 2 | 3R3B |  | + |  |  |  |  |  |  | + | + | + |  |  |  |  |  |  |  | + |  |  | + |
|  | 3R2E |  |  | + |  |  |  |  |  | + | + | + | + | + | + |  | + | + |  | + |  |  |  |
|  | 3R2D | + |  |  |  |  |  |  |  | + | + | + |  |  |  | + | + |  |  | +P |  |  | + |
|  | 3R2C | + |  |  |  |  | + |  |  | + | + | + |  |  | + |  |  | + |  | + |  |  | + |
|  | 3R1E | + |  |  |  |  |  |  |  | + |  | + | + | + |  |  | + |  |  | + |  | + | + |
|  | 3R1D |  |  | + |  |  |  |  |  | + | + | + |  |  |  |  | + |  |  | + |  |  | + |
|  | 3M2D | + |  |  |  |  |  |  |  | + | + | + | + |  |  | + | + |  |  | + | + |  |  |
|  | 3M2C | + |  |  |  |  |  |  |  | + | + | + | + |  |  |  | + |  |  | + |  |  | + |
|  | 3M2A |  | + |  |  |  |  |  |  | + | + | + | + |  |  | + | + |  |  | + |  |  |  |
|  | 3M1E |  |  | + |  |  |  |  |  |  | + | + | + |  |  |  |  |  |  | + |  |  | + |
|  | 3M1C |  |  | + |  |  |  |  |  | + |  | + |  |  |  |  |  |  |  |  |  |  | + |
|  | 3M1A |  | + |  |  |  |  |  |  | + |  | + |  |  |  |  | + |  |  |  |  |  | + |
|  | 3L2C | + |  |  |  |  |  |  |  | + | + | + | + | + | + | + | + |  |  | + |  |  |  |
|  | 3L1E |  | + |  |  |  |  |  |  | + | + | + | + |  |  |  | + |  |  |  |  |  |  |
|  | 3L1D | + |  |  |  |  |  |  |  | + | + | + |  |  |  | + | + |  |  | + |  |  |  |
|  | 3L1C | + |  |  |  |  |  |  |  | + | + | + | + |  |  | + | + |  |  | +P |  | + | + |
|  | 3L1B | + |  |  |  |  |  |  |  | + | + | + | + |  |  | + | + |  |  | + |  |  |  |
| site 3 | 6R2C | + |  |  |  |  |  |  |  | + | + | + | + |  | + | + | + |  |  | + |  |  |  |
|  | 6R2B | + |  |  |  |  |  |  |  | + | + | + | + | + |  |  | + | + |  | +P |  |  |  |
|  | 6R1E |  | + |  |  |  |  |  |  | + |  | + | + | + | + | + | + |  |  | + |  |  | + |
|  | 6R1D | + |  |  |  |  |  |  |  | + | + | + |  | + |  | + | + |  |  | +P |  |  |  |
|  | 6R1C | + |  |  |  |  |  |  |  |  | + | + | + |  | + |  | + | + |  | + |  | + |  |
|  | 6R1B |  |  |  | + |  |  |  |  | + |  | + | + |  |  |  |  |  |  | + | + | + | + |
|  | 6M3C | + |  |  |  |  |  |  |  |  | + | + | + |  |  |  | + |  |  | + |  |  |  |
|  | 6M3A | + |  |  |  |  |  |  |  | + | + | + | + |  |  | + | + |  |  | + |  | + |  |
|  | 6M2B | + |  |  |  |  |  | + |  | + | + | + | + |  |  |  |  |  |  | + |  |  |  |
|  | 6M2A | + |  |  |  |  |  |  |  | + | + | + | + |  |  | + | + |  |  | + |  |  |  |
|  | 6M1E |  | + |  |  |  |  |  |  | + | + | + | + | + | + | + | + |  |  | + |  |  |  |
|  | 6M1B | + |  |  |  |  |  | + |  |  |  | + | + | + |  |  | + | + |  | +P | + | + |  |
|  | 6M1A | + |  |  |  |  |  |  |  |  |  | + | + | + | + | + | + | + |  | +P |  | + | + |
|  | 6L2B | + |  |  |  |  |  |  |  | + | + |  | + | + | + | + | + | + |  | + | + | + |  |
|  | 6L2A |  | + |  |  |  |  |  |  | + | + | + | + | + | + | + | + | + |  | + |  | + | + |
|  | 6L1E |  | + |  |  |  |  |  |  | + | + | + |  | + | + | + | + | + |  | + |  |  |  |
|  | 6L1C |  | + |  |  |  |  |  |  | + |  | + | + | + | + | + | + |  |  |  |  |  | + |
|  | 6L1B |  | + |  |  |  | + |  |  | + |  | + | + |  | + |  | + | + |  | + |  | + |  |
|  | 6L1A | + |  |  |  |  |  |  |  |  |  | + |  |  |  | + | + |  |  | +P |  |  |  |
| site 4 | 5R2D |  |  | + |  |  |  |  |  | + | + | + | + | + | + | + | + |  |  | + |  |  |  |
|  | 5R2C |  |  | + |  |  |  |  |  | + | + | + |  | + | + | + | + |  |  | + |  |  |  |
|  | 5R2B | + |  |  |  |  |  |  |  | + |  | + | + |  | + |  |  |  |  | +P |  | + | + |
|  | 5R2A | + |  |  |  |  |  |  |  | + |  | + |  |  |  |  |  |  |  | + |  |  | + |
|  | 5R1D | + |  |  |  |  |  |  |  | + | + | + | + |  | + |  |  |  |  | + |  |  |  |
|  | 5R1C | + |  |  |  |  |  |  |  |  | + | + |  |  | + | + | + |  |  | + |  |  | + |
|  | 5M2D | + |  |  |  |  |  | + |  | + | + | + | + |  |  |  | + |  |  | + |  |  |  |
|  | 5M2C |  | + |  |  |  |  |  |  | + | + | + | + | + |  | + | + | + | + | + |  |  |  |
|  | 5M2A | + |  |  |  |  |  |  |  | + | + | + | + |  |  |  | + |  |  | + |  | + | + |
|  | 5M1A | + |  |  |  |  |  |  |  | + | + | + |  |  |  |  | + |  |  | +P |  |  | + |
|  | 5L3D | + |  |  |  |  |  |  |  | + |  | + | + |  | + |  | + |  |  | + |  |  | + |
|  | 5L3C |  | + |  |  |  |  |  |  | + | + | + | + |  |  |  | + |  |  | + |  |  |  |
|  | 5L3B | + |  |  |  |  |  |  |  |  | + | + | + |  |  |  |  |  |  | + |  |  | + |
|  | 5L2C |  | + |  |  |  |  |  |  | + | + | + | + |  | + | + | + |  |  | + |  |  |  |
|  | 5L2B | + |  |  |  |  |  |  |  | + | + | + | + |  |  |  |  |  |  | + |  |  | + |
|  | 5L2A | + |  |  |  |  |  |  |  | + | + | + | + |  |  |  |  |  |  | + |  |  | + |
|  | 5L1C | + |  |  |  |  |  |  |  | + | + | + | + | + |  |  | + |  |  | + |  |  |  |
|  | 5L1A |  |  | + |  |  | + |  |  | + | + | + | + |  |  | + | + |  |  | + |  |  | + |
| site 5 | 1R3C | + |  |  |  |  |  |  |  | + | + | + |  | + |  | + | + |  |  | + |  |  |  |
|  | 1R3B | + |  |  |  |  |  |  |  |  |  | + | + |  |  |  |  |  |  | +P |  | + | + |
|  | 1R3A |  |  |  |  | + |  |  |  |  |  | + | + | + |  |  | + |  |  | + |  |  |  |
|  | 1R2C | + |  |  |  |  |  |  |  | + |  | + | + | + | + |  | + |  |  | +P |  | + | + |
|  | 1R2B | + |  |  |  |  | + |  |  | + | + | + |  | + |  | + | + | + |  | + |  | + |  |
|  | 1R2A |  | + |  |  |  |  | + |  |  | + | + | + | + | + | + | + |  |  | + |  | + |  |
|  | 1R1D | + |  |  |  |  |  |  |  | + | + | + | + |  |  |  |  |  |  | + |  |  | + |
|  | 1R1C | + |  |  |  |  |  |  |  | + | + | + |  |  |  |  | + |  |  | +P |  |  | + |
|  | 1R1B | + |  |  |  |  |  |  |  |  |  | + |  |  | + |  | + | + |  | + |  |  |  |
|  | 1M3C | + |  |  |  |  | + |  |  | + | + | + | + |  |  |  |  |  |  | + |  |  |  |
|  | 1M2C |  | + |  |  |  |  |  |  | + | + | + | + | + |  | + | + |  |  | + |  | + | + |
|  | 1M2B |  | + |  |  |  |  |  |  | + | + | + | + |  |  |  | + |  |  | + |  | + | + |
|  | 1M1D | + |  |  |  |  |  |  |  | + | + |  |  |  | + | + | + |  |  | +P |  | + | + |
|  | 1M1B | + |  |  |  |  |  |  |  | + | + | + | + |  |  |  |  |  |  | + | + |  |  |
|  | 1M1A | + |  |  |  |  |  |  |  | + |  | + | + |  |  |  | + |  |  |  |  | + | + |
|  | 1L3D |  |  |  | + |  | + | + |  |  | + | + |  | + | + | + | + | + |  | + |  | + | + |
|  | 1L3C | + |  |  |  |  |  |  |  | + | + | + |  |  | + |  | + |  |  | +P |  |  | + |
|  | 1L3B |  | + |  |  |  |  |  |  |  | + | + |  | + |  | + | + |  |  |  |  |  |  |
|  | 1L2B |  | + |  |  |  |  |  |  | + | + | + |  |  | + |  | + |  |  | + |  |  |  |
|  | 1L2A |  | + |  |  |  |  |  |  | + | + | + | + | + | + |  |  |  |  | + |  |  |  |
|  | 1L1D | + |  |  |  |  | + |  |  | + | + | + |  | + | + |  | + | + |  | +P |  |  | + |
|  | 1L1C | + |  |  |  |  |  |  |  | + | + | + |  |  |  |  | + |  |  | + |  |  |  |
|  | 1L1B | + |  |  |  |  |  |  |  | + | + | + | + |  |  |  | + |  |  | + |  | + |  |
|  | 1L1A | + |  |  |  |  |  |  |  | + | + | + | + |  |  |  | + |  |  | +P |  | + | + |

+P denotes enterococci isolates with phenotypically active gelatinase determinant
